# Prediction of genetic value for sweet cherry fruit maturity among environments using a 6K SNP array

**DOI:** 10.1038/s41438-018-0081-7

**Published:** 2019-01-01

**Authors:** Craig M. Hardner, Ben J. Hayes, Satish Kumar, Stijn Vanderzande, Lichun Cai, Julia Piaskowski, José Quero-Garcia, José Antonio Campoy, Teresa Barreneche, Daniela Giovannini, Alessandro Liverani, Gérard Charlot, Miguel Villamil-Castro, Nnadozie Oraguzie, Cameron P. Peace

**Affiliations:** 1 University of Queensland, Queensland Alliance for Agriculture and Food Innovation, Brisbane, QLD 4072 Australia; 2 The New Zealand Institute for Plant and Food Research Limited, Hawke’s Bay Research Centre, Hastings, 4130 New Zealand; 3 Department of Horticulture, Washington State University, Pullman, WA 99164 USA; 4 Department of Horticulture, Michigan State University, East Lansing, MI 48824 USA; 5 UMR 1332 BFP, INRA, University of Bordeaux, 33140 Nouvelle-Aquitaine, France; 6 Council for Agricultural Research and Economics (CREA), Fruit Unit of Forlì, Via la Canapona, 1 bis, 47121 Emilia-Romagna, Italy; 7 Centre Technique Interprofessionnel des Fruits et Légumes (CTIFL), 751 Chemin de Balandran, 30127 Bellegarde, France; 8 Department of Horticulture, Washington State University, Irrigated Agriculture Research and Extension Center, 24106N Bunn Road, Prosser, WA 99350 USA

**Keywords:** Agricultural genetics, Plant breeding

## Abstract

The timing of fruit maturity is an important trait in sweet cherry production and breeding. Phenotypic variation for phenology of fruit maturity in sweet cherry appears to be under strong genetic control, but that control might be complicated by phenotypic instability across environments. Although such genotype-by-environment interaction (G × E) is a common phenomenon in crop plants, knowledge about it is lacking for fruit maturity timing and other sweet cherry traits. In this study, 1673 genome-wide SNP markers were used to estimate genomic relationships among 597 weakly pedigree-connected individuals evaluated over two seasons at three locations in Europe and one location in the USA, thus sampling eight ‘environments’. The combined dataset enabled a single meta-analysis to investigate the environmental stability of genomic predictions. Linkage disequilibrium among marker loci declined rapidly with physical distance, and ordination of the relationship matrix suggested no strong structure among germplasm. The most parsimonious G × E model allowed heterogeneous genetic variance and pairwise covariances among environments. Narrow-sense genomic heritability was very high (0.60–0.83), as was accuracy of predicted breeding values (>0.62). Average correlation of additive effects among environments was high (0.96) and breeding values were highly correlated across locations. Results indicated that genomic models can be used in cherry to accurately predict date of fruit maturity for untested individuals in new environments. Limited G × E for this trait indicated that phenotypes of individuals will be stable across similar environments. Equivalent analyses for other sweet cherry traits, for which multiple years of data are commonly available among breeders and cultivar testers, would be informative for predicting performance of elite selections and cultivars in new environments.

## Genetics: Sweet news for cherry breeders Or Genetics: Cherry-ripening is predictable in new environments

The fruiting season of a cherry is short and sweet, but at least it’s relatively consistent across different environments, a new genetic analysis suggests. The development of additional early- and late-maturing sweet cherry cultivars is a major objective for cherry-breeders, but the influence of the environment on the timing of fruit maturity—which could reduce the accuracy of selective breeding efforts - was unclear. So, Craig Hardner at the University of Queensland in St Lucia and colleagues used DNA markers to  model relationships among individuals and examined the dates at which their fruit ripened at four different locations in Europe and the USA across two seasons. The timing of fruit maturity was relatively stable between related individuals across similar environments, suggesting that new cherry cultivars could be developed without having to test them at multiple sites.

## Introduction

The timing of fruit maturity is important for sweet cherry (*Prunus avium* L.) production, particularly given the brief shelf life of the fruit. Fruit of an individual tree tends to ripen over a narrow window of 3–7 days and firmness and taste decline rapidly over 4–7 days after harvest, even under cold storage^1–4^. Similarly, the harvest window for cherry is relatively short^5–7^. Fruit produced in the early or later part of the production season attracts higher prices than in the mid-season^8^.

There is evidence that phenotypic variation in phenology of fruit maturity in sweet cherry is under strong genetic control, suggesting breeding opportunities for this trait. Traditionally, sweet cherry cultivars are divided according to their maturity date into early (e.g., ‘Burlat’ and ‘Chelan’), mid- (e.g., ‘Bing’) and late season (e.g., ‘Sumtare’)^9^. Individual broad-sense heritability of fruit maturity timing is high (0.76–0.83)^10,11^ and large-effect Quantiatative Trait Loci (QTLs) on linkage groups 1, 4, 5, and 6 have been detected within a full-sib family (‘Regina’ × ‘Lapins’), each explaining up to 20% of phenotypic variation^10,12^.

While cherry has been cultivated for more than 2000 years, breeding commenced around the early 1800s^13,14^. From the early 20th century, modern breeding involving selection of elite parents was undertaken in the USA and Canada, followed by European countries such as the UK, Russia, Ukraine, and others by the 1950s. In the last 30 years, breeding has progressed rapidly, fuelled in part by the adoption of dwarfing rootstocks allowing intensive orchard production^15^. Sansavini and Lugli^16^ noted that during the period 1991–2004, 230 new cultivars were released, this number being second in stone fruit only to peach. Nevertheless, some very old selections, such as ‘Burlat’ or ‘Bing’, and cultivars with unclear origin such as ‘Ambrunés’, ‘Emperor Francis’, ‘Germersdorfer’, ‘Hedelfingen’, ‘Napoleon’ or ‘0900 Ziraat’, are still widely planted. Cherry breeding has relied on a narrow genetic base for the main founders^17^, and modern cultivars are only a few generations removed from early ancestors^18^.

The development of early- and late-maturing cultivars is a major objective in sweet cherry breeding across the globe^10,13^. Replacing ‘Burlat’ has been an important target for decades, although most new cultivars of the same early maturity period have undesirable fruit quality in terms of firmness, tolerance to rain-induced fruit cracking, or post-harvest shelf life. The breeding program conducted at Summerland, Canada, has released several commercially successful late-maturing cultivars, such as ‘Sumtare’ (Sweetheart™), ‘13S2009’ (Staccato™) and ‘13S2101’ (Sovereign™).

Genotype-by-environment interaction (G × E) is a common phenomenon in crop plants that can complicate selection^19,20^. Confirming the degree of G × E is an important initial step in developing strategies for commercial deployment of improved germplasm in target production environments^19,21^. If the degree of G × E is small, germplasm can be selected for overall mean performance across the target region. Alternatively, if the magnitude of G × E is large and repeatable factors can be identified that explain some of the pattern in G × E, higher gains can be achieved by using those factors to subdivide production regions into ‘mega-environments’ and select germplasm targeted for each. Otherwise, the presence of G × E represents experimental noise, reducing selection accuracy.

Detection of G × E in plants requires replication of genetic effects across multi-environment trials (METs). Commonly, METs in horticulture are established with individuals that are clonally replicated across environments, e.g., refs. ^22–25^, or are within related families connected through a pedigree structure that is used to estimate an expected relationship matrix^26^. Thereby, the performance of an individual in one environment can be genetically correlated with its performance in other environments^27,28^ and that correlation can be used to predict the performance of genetic treatments (e.g., cultivars, or even specific alleles) in environments in which they have not been directly tested^29^. However, conducting conventional METs in horticultural tree crops is expensive, particularly due to the large size of experimental units and long juvenility periods. As a consequence, tree fruit breeding and elite selection or cultivar evaluation programs are often localised and have limited replication of germplasm among programs. These features limit the opportunity to evaluate G × E for horticultural tree crops.

Linear mixed models are the preferred approach for analysing data from METs^30^. Such models support estimation of variance and covariances to describe G × E, incorporation of complex models that might include different experimental designs among trials or spatial effects, and prediction of unbiased genetic values from unbalanced data. A common model used to describe G × E from METs is a simple main effect and homogeneous interaction (G + G × E) linear mixed model. However, this model is restrictive as it unrealistically assumes a common genetic variance across environments and a common covariance (and hence correlation) among all pairs of environments^30^. More general models allow greater flexibility, although a highly unstructured covariance matrix might be difficult to estimate, particularly when the number of dimensions is large and the data for each dimension are limited^31–33^. The interaction term in the simple G + G × E model is an average of the individual pairwise covariances in the general model^34^ A parsimonious alternative is the FA parameterisation of the genetic-by-environment covariance matrix, which estimates environmental loadings for a reduced number of hypothetical orthogonal dimensions that maximise differences among environments (*λ*), and estimates specific variances for each environment for genetic effects not accounted for by the loadings (*ψ*)^31^.

Genetic relationships among individuals can be described using a DNA marker-based genomic relationship matrix (GRM)^35^, to approximate identity-by-descent as replication of chromosome segments among individuals deployed across METs. An advantage of the GRM is that it can describe heterogeneity of realised relationships within families that occur as a consequence of Mendelian sampling^36^. A realised relationship matrix is expected to be more accurate than a pedigree-derived relationship matrix, because the latter assumes a mean expected relationship among relatives. In addition, realised relationships might capture cryptic or undocumented relationships^37,38^. The GRM could therefore be used to replace the pedigree-derived relationship matrix to study G × E. Considerations in using the GRM with a factor analytic (FA) parameterisation of the genetic-by-environment covariance matrix to model G × E have been described previously^39^.

The aim of this study was to use a G × E model involving a GRM to combine phenotypic data on fruit maturity timing collected in multiple locations for cherry germplasm having little clonal replication among locations, to thereby estimate patterns in G × E and predict performance of individuals in environments in which they have not been tested. By leveraging the information from existing breeding program datasets in this way, we hypothesised achieving improved accuracy of genetic predictions within and across locations compared to predictions arising from datasets of single breeding programs.

## Methods

### Description of data

**Fig. 1:**
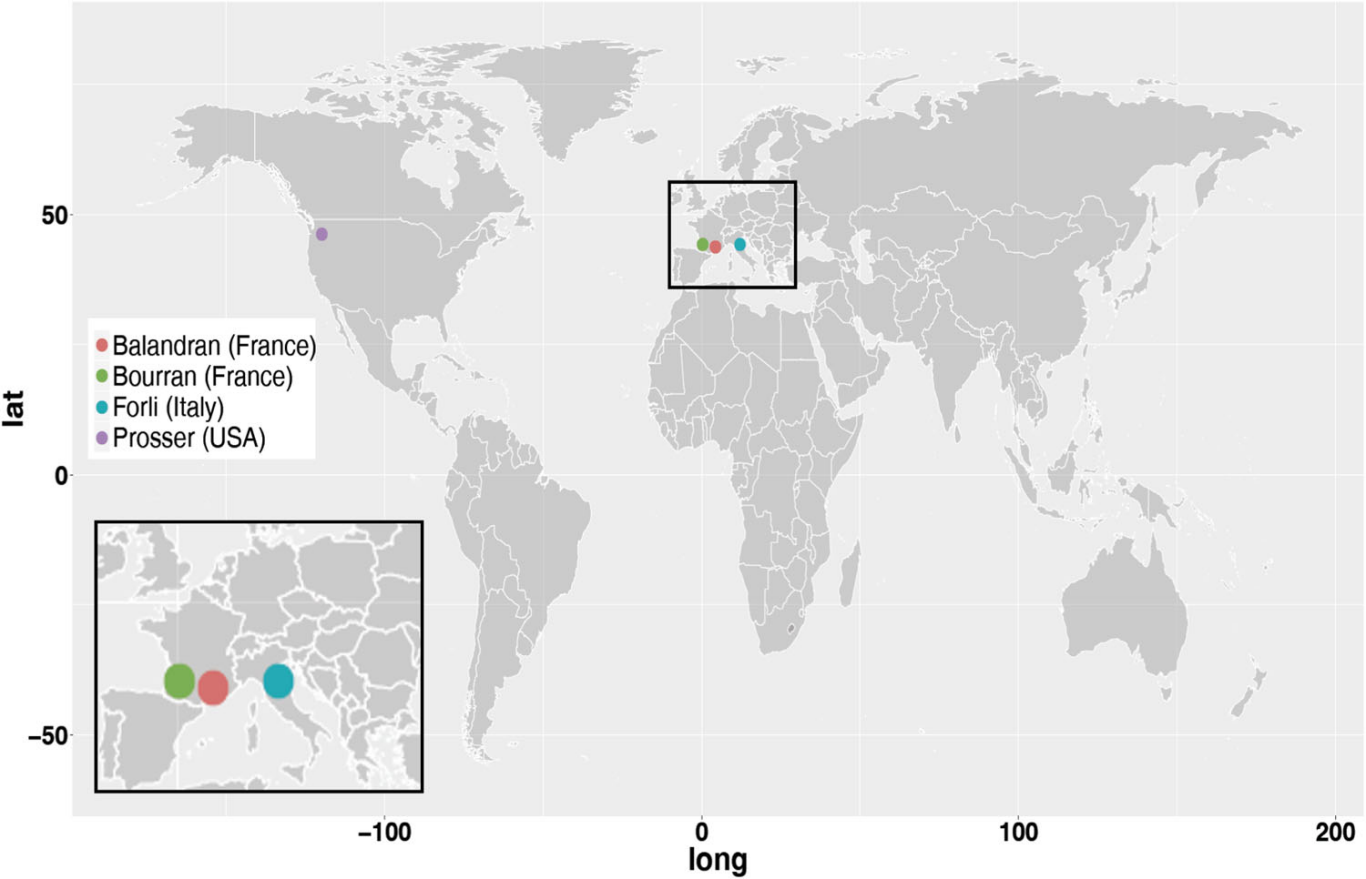
Location of field trials included in this study

**Fig. 2:**
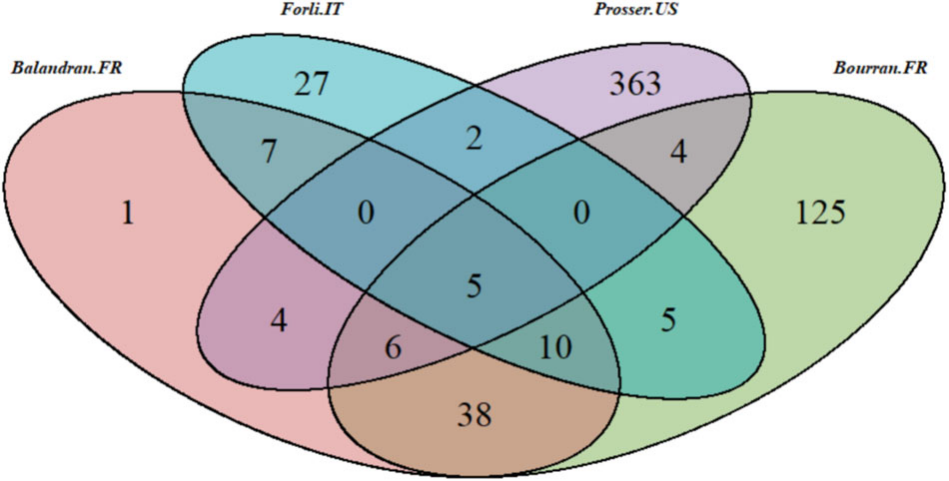
Venn diagram of numbers of individuals specific to and shared among four trial locations. FR France, IT Italy, US USA

Germplasm used consisted of 597 sweet cherry cultivars, selections and unselected offspring, subsets of which were grown at four locations: Prosser, WA, USA (46.291383, −119.746753); Bourran (44.333793, 0.413504) and Balandran (43.757312, 4.461919), France; and Forlí (44.216667, 12.05000), Italy (Fig. 1). Fruit maturity timing was assessed at each location for two seasons (Table 1) to give eight unique location-by-season environments. Most individuals at Balandran were also assessed at Bourran, but there was less overlap among the individuals in France and those at the Italian and Prosser locations, and only five individuals were present at all four locations (Fig. 2).

**Table 1:** Summary of the phenotypic dataset of fruit maturity timing from eight location-by-season environments

Trial	Location	Country	Trait	Season	No. of individuals	Mean^a^	Variance^a^
Balandran.FR	Balandran	France	Fruit maturity date	1997	61	144	103.8
Balandran.FR	Balandran	France	Fruit maturity date	1998	50	148	100.5
Bourran.FR	Bourran	France	Fruit maturity date	2014	187	150	81.3
Bourran.FR	Bourran	France	Fruit maturity date	2015	192	150	74.6
Forlí.IT	Forlí	Italy	Date of first harvest	2014	50	149	141.0
Forlí.IT	Forlí	Italy	Date of first harvest	2015	55	152	122.5
Prosser.US	Prosser	USA	Fruit maturity date	2011	231	186	60.1
Prosser.US	Prosser	USA	Fruit maturity date	2012	360	184	50.6

^a^Units are in Julian days

The 71 individuals assessed at Balandran, France (trial ‘Balandrin.FR’), belonged to sweet cherry trials of the ‘Chart of evaluation of fruit varieties’ program coordinated by Centre Technique Interprofessionnel des Fruits et Légumes (CTIFL) and involving Institut National de la Recherche Agronomique (INRA), regional experimental stations, representatives from growers associations, and nurserymen and private breeders from France and beyond. Balandran is one of the three locations in France where new cultivars are initially evaluated and is 44 m above sea level. The climate is classified as Mediterranean and the trial was planted on Costiéeres soil. Each cultivar was grafted onto rootstock ‘Colt’ and planted as two clonal replicates, adjacent wherever possible. Trees were trained to a vase with four to five permanent branches. The trial was irrigated and managed using standard approaches. Fruit maturity timing was measured as the date (in Julian days) on which 50% of the fruit on both accessions were mature.

The 193 individuals assessed at Bourran, France (trial ‘Bourran.FR’) were accessions of a germplasm collection maintained by the *Prunus* Genetic Resources Center of INRA at Bourran (Lot & Garonne), near Bordeaux^40^. The site is 50 m above sea level. The climate is classified as oceanic and the soil type is clay alluvium. Two replicate trees for each individual on the rootstock ‘Maxma 14′ were planted adjacently in a random design. Approximately half were of French breeding origin and the remainder were from 15 other countries in North America, Asia and Europe. Accessions were historic landrace cultivars (*n* = 85) and recently bred cultivars currently planted in Europe (*n* = 106). Two accessions of each of the cultivars Noir d’Ecully and Giorgia were also included, each corresponding to a different period of introduction into the INRA collection. Trees were trained to an open vase and were irrigated and managed using a standard commercial regime. Fruit maturity timing was measured as the date (in Julian days) on which at least 70% of the fruit on both replicate trees were mature, using firmness, taste and skin colour indicators.

The pool of 56 individuals assessed at Forlí, Italy (trial ‘Forlí.IT’), was composed of traditional germplasm from the Emilia-Romagna territory, modern cultivars from various breeding programs, breeding selections, and a few clones of known cultivars selected in the 1970s after irradiation of bud sticks and are part of the sweet cherry collection at CREA (Council for Agricultural Research and Economics)—Forlì. The site is 34 m above sea level, with Mediterranean climate and silty-clay soil with medium-low organic matter soil. Each individual was represented by two to three adjacent replicate trees grafted onto the rootstock ‘Colt’. Trees were managed according to integrated production practices, irrigated and trained to a vase. Fruit maturity timing was measured as the date (in Julian days) that the first 10% of fruit on all replicate trees were ripe for consumption^41^ using firmness, taste and skin colour indicators.

The 384 individuals evaluated at Prosser, USA (trial ‘Prosser.US’) were within the RosBREED sweet cherry Crop Reference Set (*n* = 268)^42^ and the Pacific Northwest Sweet Cherry Breeding Program at Washington State University. The site is ~200 m above sea level. The climate is classified as semi-arid and the soil is well-drained non-saline from the Aridisols order. The RosBREED Crop Reference Set consists of cultivars, founders, ancestors, advanced selections and unselected offspring representing germplasm used in US breeding programs^42,43^. A total of 53 Prosser.US individuals were cultivars or breeding selections, with the remainder consisting of unselected offspring from 98 families (53 families with two or more offspring). The germplasm was planted between 2006 and 2008 as single trees of each individual, with offspring often arranged in families. Trees were managed using conventional orchard management practices. All individuals were grown on ‘Gisela 6’ rootstock except for own-rooted offspring. Fruit maturity timing was measured in 2011 and 2012 within the RosBREED project^44^ as the date (in Julian days) at which 50% of fruit on a tree were mature using firmness, taste and skin colour indicators^43^.

SNP data generated using the RosBREED cherry 6K SNP array v1^45^ were available from other projects for the 597 individuals from the four locations described above (Fig. 2). Briefly, DNA samples had been extracted using standard methods, e.g.^40^, and run on the array following manufacturer’s instructions. Resultant probe intensity data were used as input for GenomeStudio to obtain genotype calls for each SNP of each individual^46^. For SNP data available from RosBREED^45^, CREA and CITA (Centro de Investigación y Tecnología Agroalimentaria de Aragón, Spain), GenomeStudio results were exported and used as input to the software program ASSIsT^47^ to obtain a subset of robust SNPs with reliable genotype calls. A multi-step pipeline using FlexQTL, VisualFlexQTL^48^ and Pedihaplotyper^49^ to detect genotyping errors was applied to obtain a high-quality genotypic dataset of 1617 SNPs. Data for 1215 high-quality SNPs loci were available from INRA, and for 1636 SNPs from CREA.

A data-curation pipeline was developed to manage germplasm, SNP and phenotypic data contributed from the various sources. The first step in the process was standardisation of names of individuals common across datasets. Initially, the format of an individual’s name was simplified by removing special characters, changing all upper case letters to lower case, and inserting an underscore between characters separated by a space. Formatted names were then compared to an existing dictionary of original names, formatted names and final names (which were the formatted final names used to link data in cases of multiple sources of data for the same individual) created from previous data sources. Full and partial matching were used to identify potential matches in the existing dictionary. New names that could not be reconciled were added to the dictionary.

Curation of SNP data involved standardisation of SNP names across the datasets against NCBI standard identities. SNP data from individuals common to multiple locations were compared to ensure consistency and fill in missing genotypic calls. The very few inconsistencies in genotypes among common individuals were set to missing, except in one case: ‘Noire de Meched’ genotyped by INRA and found to be substantially different to an individual with the same name in the CREA collection (the former believed to be the original, true-to-type cultivar introduced from Iran). In this case, cultivar names were modified to distinguish the difference. SNP loci with missing data of >30% or a minor allele frequency <0.05 were removed, leaving 1273 loci for 550 individuals with unique genome-wide DNA profiles (i.e., 47 of the original 597 individuals were determined to be duplicate genotypes). The missing 33,704 (4% of the total) individual SNP locus genotypes were imputed with a hidden Markov Model implemented in Beagle^50,51^ in the R package synbreed^52^ using only flanking markers, i.e., without considering family information.

Phenotypic data curation began with incorporation of standardised individual names. Next, field names in original data files that identified experimental and sampling design factors were identified and renamed using standardised terms (Table 2). A field representing the experimental unit in each trial was created to identify the smallest unit to which a unique random genetic treatment was applied^53^. Fields not in the list of standard design and sampling effects were retained. Phenotypic data was reshaped to long format (i.e., each record represented an individual observation).

**Table 2:** Standardised field terms for use in combining phenotypic datasets

Field	Description
Location	Spatial unit containing one or more trials for which genetic effects are assumed to be homogeneous (i.e., homogeneous genetic variance and genetic correlation of 1)
Trial	Spatial unit that contains experimental units grouped by a particular factor (usually planting date)
Section	Spatial unit within location for which experimental units are contiguous
Row	Spatial coordinate within section that indexes the distribution of experimental unit along the planting row dimension
Position	Spatial coordinate within section that indexes experimental unit along dimension perpendicular to planting row
Block	Design unit within section that indexes group of experimental units grouped to account for spatial variation
Plot	Design unit within block that indexes group of experimental units grouped to account for spatial variation
Plant	Individual unit that represents unique propagation event
Planting date	Date at which experimental unit was established
REF_ID	Standardised genetic treatment
Experimental unit	Unit at which genetic treatment is applied, i.e., unit on which sampling may be temporally repeated. Created as the unit combination of design units and genetic treatment
Year	Year at which observation is made
Observation unit	Created as unit combination of experimental unit and factor defining repeated observations on experimental unit
y	Untransformed observation

### Statistical methods

A multivariate genomic prediction linear mixed model was implemented to estimate model parameters and predict fixed and random effects for fruit maturity data collected from individuals planted across the four locations and assessed over Table 3 two seasons, i.e., in eight location-by-season environments. The general mixed model for predicting genomic breeding values of *n*_G_ individuals, evaluated at *n*_L_ locations and *n*_Sj_ seasons within the *j*^th^ location, was:

$${\bf{y}} = {\bf{Xb}} + {\bf{Zu}} + {\bf{r}},$$

where **y** was a vector of observations, **b** was vector of unknown fixed effects (mean effect of location-by-season environment, and year of planting at the Balandran location—the only location where this factor was relevant), **X** was the design matrix for fixed effects, **u** was a vector of unknown random effects (genomic breeding value for each of the *n*_G_ individuals, at each of the *n*_E_ location-by-season environments), **Z** was the design matrix for random effects and **r** was a vector of unknown residual effects, with variation of observations assumed to follow a multivariate normal distribution:

$${\mathrm{var}}\left( {\bf{y}} \right) = {\bf{ZGZ}}^T + {\bf{R}},$$

where **G** was the variance–covariance of additive genomic effects for the *n*_G_ individuals among the *n*_E_ location-by-season environments and **R** was the variance–covariance among residual effects.

**Table 3:** Estimates of additive genomic variation (vG), residual variation (vR), phenotypic variation (vP) and narrow-sense heritability (*h*^2^) for eight location-by-season environments

Location	Season	vG	vR	vP	*h* ^2^
Balandran.FR	1997	111.3	13.8	125.1	0.89
Balandran.FR	1999	101.4	10.5	111.9	0.91
Bourran.FR	2014	60.3	12.7	73.0	0.83
Bourran.FR	2015	57.1	8.1	65.2	0.88
Forlí.IT	2014	110.4	19.3	129.7	0.85
Forlí.IT	2015	98.4	20.0	118.4	0.83
Prosser.US	2011	27.6	18.1	45.7	0.60
Prosser.US	2012	26.5	17.6	44.1	0.60

The (*n*_G_ × *n*_E_) × (*n*_G_ × *n*_E_) matrix **G** was separated into the *n*_G_ × *n*_G_ additive genomic relationship matrix (**G**_*A*_), estimated following VanRaden^35^ method 1 using the complete imputed individual-by-SNP matrix and the *n*_E_ × *n*_E_ additive genetic-by-environment covariance matrix (**G**_A×E_), i.e.:

$${\bf{G}} = {\bf{G}}_{\mathrm{A}} \otimes {\bf{G}}_{{\mathrm{A \times E}}},$$

where ⊗ was the Kronecker product matrix operation. The *n*_E_ × *n*_E_ additive genomic-by-environment covariance matrix, **G**_A×E_, can be modelled using various structures^30^. Assuming homogeneous genomic variances across all environments and perfect genomic correlations (i.e., *r*_A_ = 1) among all pairwise environmental combinations leads to ${\bf{G}}_{{\mathrm{A \times E}}} = \sigma _{\mathrm{A}}^2 \otimes {\bf{1}}_{n{\mathrm{E}}}$, where **1**_*n*E_ was a *n*_E_ × *n*_E_ matrix of 1 s. A compound structure, i.e., homogeneous variances on the diagonal and homogenous covariances on the off-diagonals, is equivalent to a main effect interaction model (i.e., A + A × E)^30^. Alternatively, a FA parameterisation models **G**_A×E_ as a reduced set of parameters that are environmental loadings (${\bf{\Lambda }}_{\mathrm{{A}} \times {{\mathrm{E}}}}$) on hypothetical orthogonal environmental dimensions that explain the maximum variation at each observed environmental dimension and environment-specific variation not explained by the loadings (**Ψ**_A×E_). In this study, only a first-order FA model was examined. The variance–covariance matrix of residual effects (**R**) was modelled as independent blocks with each block representing the variance–covariance among observations across the seasons at the *j*^th^ location (**R**_*j*_), which was separated into **R**_Sj_ (residual-by-season covariance matrix for the *j*^th^ location) and **I**_nj_ (the correlation matrix of residuals, an identity matrix of dimensions nj, the number of experimental units evaluated at the *j*^th^ location).

Parameters of the random and residual models were estimated using average information approaches implemented in the statistical software ASReml^54^. Independent analyses for each specific location-by-season environment were undertaken to review the distribution of residuals. Best linear unbiased estimates (E-BLUEs) of fixed effects and best linear unbiased predictions (E-BLUPs) of random effects (breeding value) were produced following Henderson’s^55^ mixed model equations using estimated **G** and **R** matrices.

The pattern of linkage disequilibrium across the experimental population was examined by plotting individual pairwise squared correlations of SNP locus genotypes among individuals (*ρ*^2^) estimated using the software PLINK^56^ against pairwise physical distances. Average linkage disequilibrium (LD) decay in physical distance was converted to genetic distance using the map presented in Guajardo et al.^57^

An eigen-value decomposition of the individual-by-location GRM (**C**) was undertaken to examine the germplasm genetic structure across the four locations. The matrix **C** was estimated as:

$${\bf{MG}}_{\mathrm{A}}{\bf{M}}^T$$

where **M** was an *m* × *n*_G_ incidence matrix (where *m* was the number of unique individual-by-location realisations and *n*_G_ was previously defined) that mapped the incidence of an individual onto specific individual-by-environment realisations. A pedigree-derived additive genetic relationship matrix was estimated from known pedigree records to examine the similarity between expected (pedigree-derived) and estimated realised GRMs.

The average additive genomic correlation among the eight location-by-season environments was estimated as:

$${\bar{r}}_{{{{\rm{A}} \times {\rm{E}}}}} = \frac{{\sigma _{\mathrm{A}}^2}}{{\sigma _{\mathrm{A}}^2 + \sigma _{{{{\rm{A}} \times {\rm{E}}}^2}}}},$$

where $\hat \sigma _{\mathrm{A}}^2$ was the off-diagonals of the compound symmetry **G**_A×E_ structure, or the variance of the additive genetic main effect across environments for the A + A × E model, and $\hat \sigma _{\mathrm{{A \times E}}}^2$ was the difference between the diagonal and the off-diagonals, or the average additive genomic-by-environment variance.

The additive genomic-by-environment covariance matrix of the FA model was estimated as:

$$\widehat {\bf{G}}_{\mathrm{{A \times E}}} = \widehat {\bf{\Lambda }}_{\mathrm{{A \times E}}}\widehat {\bf{\Lambda }}_{\mathrm{{A \times E}}}^T + \widehat {\bf{\Psi }}_{\mathrm{{A \times E}}},$$

where $\widehat {\bf{\Lambda }}_{\mathrm{{A}} \times {\mathrm{E}}}$was the *n*_E_ × *k* matrix of estimated loadings for the hypothetical orthogonal factor for the additive genomic variance at the *n*_E_ environments, and $\widehat {\bf{\Psi }}_{\mathrm{A}}$ is a *n*_E_ × *n*_E_ diagonal matrix of environment-specific variances.

Individual narrow-sense genomic estimated heritability for the *k*^th^ season at the *j*^th^ location was estimated as:

$$\hat h_{jk}^2 = \frac{{\hat \sigma _{{\mathrm{A}}_{jk}}^2}}{{\hat \sigma _{{\mathrm{A}}_{jk}}^2 + \hat \sigma _{{\mathrm{R}}_{jk}}^2}},$$

where $\hat \sigma _{{{\mathrm{A}}}_{jk}}^2$ and $\hat \sigma _{{\mathrm{R}}_{jk}}^2$ were the estimated additive genomic and residual variances for each of the *n*_E_ location-by-season environments. Genomic breeding values for fruit maturity timing were predicted using estimated parameters from the FA model.

Two cross-validation scenarios were used to estimate prediction accuracy (PACC). Firstly, a within-location validation population was created by subsampling individuals within locations, as the main interest of this study was performance predictions of individuals in environments in which they have not been tested, but phenotypes of other genotyped individuals have been used to characterise the environment of interest. Five-fold validation within locations was used. Adjusted phenotypes were obtained by removing from the phenotypic observations the effects of location, season, location-by-season, and year of planting at the Balandran location estimated from the fit of the FA model to the full dataset. Individuals at each location were then randomly assigned to one of the five sets to define location-by-set validation populations. For each validation analysis, adjusted phenotypes for the individuals in the particular location-by-set validation population were set to missing and parameters for the FA A × E model were re-estimated using the training population and then used to predict additive genomic values for the validation population. Predictive ability (PA) was estimated as the correlation between the genomic predicted breeding values for the particular location-by-set validation population and the original adjusted phenotypes. Prediction accuracy was estimated for the particular location-by-set validation population by dividing PA by the square-root of the individual narrow-sense genomic estimated heritability of genomic values from the fit of the FA A × E model to the full dataset, as independent heritability estimates were unavailable. Standard error of PACC was estimated as $1{\mathrm{/}}\sqrt {n_{{\mathrm{G}} \times {\mathrm{Lk}}}},$ where *n*_G×Lk_ was the number of individuals in the location-by-set validation population^58^.

A fourfold cross-validation was undertaken for a second scenario of dropping out the complete phenotypic data from one entire location for each validation iteration. Predictive ability was estimated as the correlation among the adjusted phenotypic performance of individuals in the removed location and the mean of the predicted breeding values for these individuals across the three preserved locations. Prediction accuracy was again estimated using genomic narrow-sense heritability of the removed environments estimated from the fit to the full dataset.

## Results

### Structure of germplasm

**Fig. 3:**
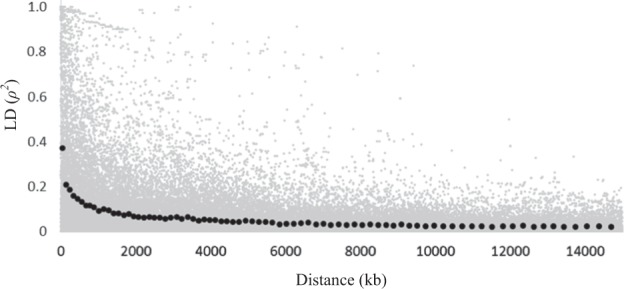
Linkage disequilibrium (LD) by physical distance (kb) for individual loci (grey dots) and averaged for each set of 1000 SNP pairs ranked according to the distance between SNPs (black dots)

The average LD between SNPs within a 100-kb window was 0.36 with the value for 20% of pairs being >0.80 (Fig. 3). There was a rapid decay in LD with distance within 250–500 kb with observed *ρ*^2^-value <0.2 between 140 and 240 kb, which corresponds to ~0.30 and 0.52 cM. At 1 cM/465 kb, LD declined to <0.14.

**Fig. 4:**
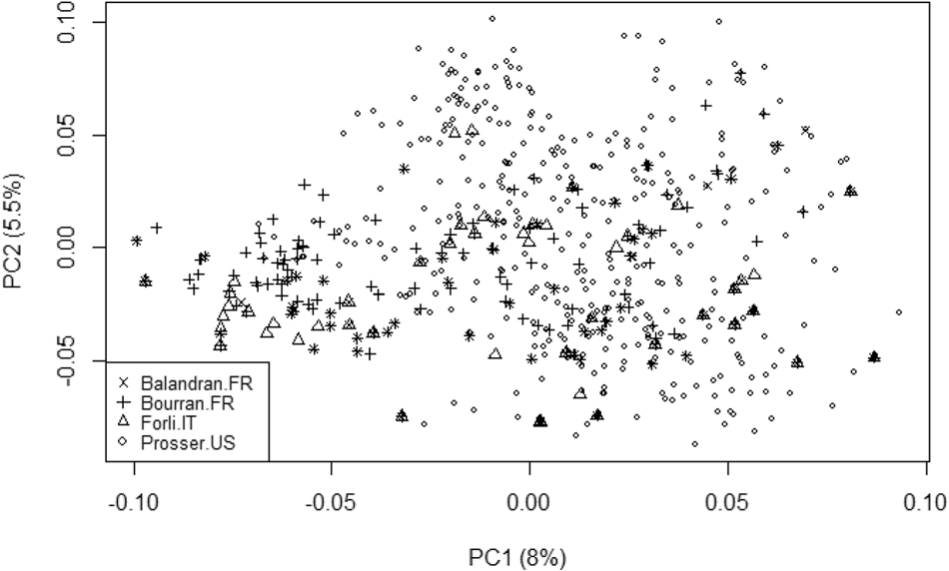
Plot of the first two vectors from an eigen decomposition of the individual-by-location genomic relationship matrix by testing location

**Fig. 5:**
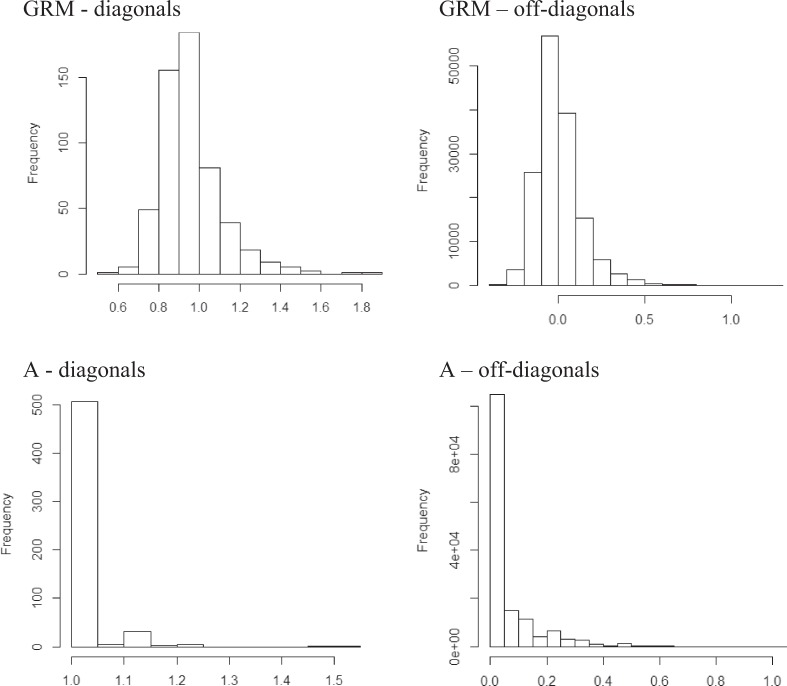
Diagonal and off-diagonal estimated realised (SNP-derived, GRM) and expected (pedigree-derived, A) additive relationship matrices elements for germplasm evaluated in this study

The first two vectors of an eigen decomposition of the estimated individual-by-location GRM explained 12% of the variation in the matrix (Fig. 4). No obvious structure was apparent in the overall relationships among and within germplasm assessed at the four locations. Distributions of elements of the relationship matrix estimated from pedigree information were quite different from the distributions for realised relationships estimated using SNP data (Fig. 5). While the mode for the distribution of diagonal elements for both the pedigree- and SNP-derived relationship coefficients was one, and 0 for the off-diagonals, the distributions of pedigree-derived coefficients were highly skewed towards these values, in contrast to the more balanced distribution of GRM coefficients (both diagonal and off-diagonal) around the mode. The highest value for the pedigree-derived diagonals was 1.5, compared to 1.8 for the estimated realised relationship coefficients. The variance of diagonal elements was greater for the GRM matrix (0.0228) compared to the pedigree-derived relationship matrix (0.0024), as was the case for the off-diagonals (0.0160 compared to 0.0103).

### Model fit

The compound symmetry A + A × E model (log-likelihood = −2554.2) was a significantly (*p* < 0.001) better fit to the data than a model that did not account for A × E (logl = −2560.7). Estimated average $\bar r_{{\mathrm{A}} \times {\mathrm{E}}}$ was 0.96. The FA A × E model (logl = −2519.2) was a significantly better fit (*p* < 0.001) than the compound symmetry (A + A × E) model.

**Fig. 6:**
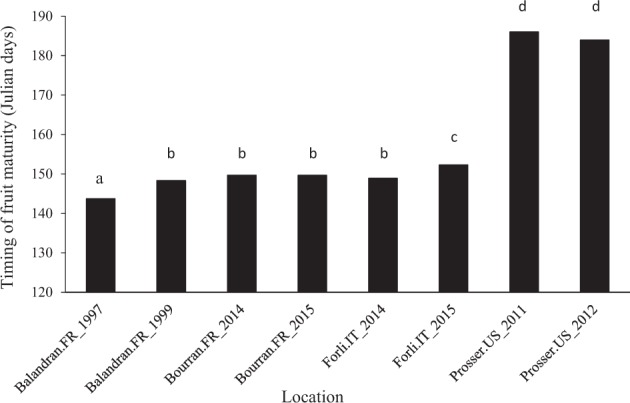
Least square means of location-by-season effects from most parsimonious G × E model. Environments with the same letter were not significantly different (*p* = 0.05)

Best linear unbiased estimation of the location-by-season environment effects for fruit maturity timing using the FA A × E model (Fig. 6) indicated that on average, and after accounting for unbalanced genetic treatments across environments, fruit maturity was significantly earlier at Balandran, France, in 1997 (144 Julian days) than all other environments. However, there was little absolute difference among the European environments, averaging <9 days. In contrast, average timing of fruit maturity at Prosser USA differed only by 2 days between year, but was around 36 days later than in Europe.

Estimated phenotypic variance differed among locations, but was more consistent between seasons within locations (Table 3). The most variable locations were Balandran and Forlí, with Prosser the least. The heterogeneity in phenotypic variance was mostly a consequence of heterogeneity in estimated additive genomic variance, particularly the lower additive genetic variance at Prosser. Estimates of individual narrow-sense heritability were >0.83 for all European environments, but lower (0.60) for the Prosser environments.

**Table 4:** Estimates of additive genomic correlation of fruit maturity timing (Julian days) among eight location-by-season environments from the fit of a FA A × E model using a genomic relationship matrix

		Balandran.FR		Bourran.FR		Forlí.IT		Prosser.US	
		1997	1999	2014	2015	2014	2015	2011	2012
Balandran.FR	1997		1	1	0.98	1	1	0.89	0.92
	1999			1	0.98	1	1	0.89	0.92
Bourran.FR	2014				0.98	1	1	0.89	0.92
	2015					0.98	0.98	0.87	0.90
Forlí.IT	2014						1	0.89	0.92
	2015							0.89	0.92
Prosser.US	2011								0.82
	2012								

Estimated additive genomic correlations among the European locations and between years within these locations from a FA A × E model with the GRM were close to 1 (Table 4). In contrast, the lowest estimated additive genomic correlation was between seasons at the Prosser location (*r*_A_ = 0.8). Estimates of the correlations between the Prosser and European environments were between 0.87 and 0.92.

### Accuracy

The overall mean genomic predictive ability of using genome-wide SNP information from phenotyped individuals at a particular location to predict performance of individuals untested at that location was 0.81, with mean PACC 0.91 ± 0.24 (Table 5, within location). Predictive ability of individual location-by-set-by-season validation populations ranged from 0.60 to 0.98, with individual PACC between 0.65 ± 0.30 to 1.06 ± 0.15 (full results not presented). Predictive ability averaged across sets within seasons was positively correlated (*r* = 0.80, *n* = 8) with estimated narrow-sense heritability.

**Table 5:** Predictive ability, prediction accuracy and standard error of prediction accuracy by location and season estimated from within-location and across-location validation

Location	Season	Within location					Across location				
		*n*G × L	*n*G × LS	PA	PACC	se.PACC	*n*G × L	*n*G × LS	PA	PACC	se.PACC
Balandran.FR	1997	14	12	0.86	0.90	0.29	63	61	0.67	0.70	0.13
	1999		10	0.88	0.91	0.32		50	0.76	0.79	0.14
Bourran.FR	2014	38	37	0.81	0.89	0.16	193	187	0.59	0.65	0.07
	2015		38	0.84	0.90	0.16		192	0.64	0.68	0.07
Forli.IT	2014	11	10	0.81	0.88	0.32	56	50	0.84	0.91	0.14
	2015		11	0.79	0.86	0.30		55	0.86	0.94	0.13
Prosser.US	2011	76	46	0.79	1.02	0.15	384	231	0.43	0.55	0.07
	2012		71	0.73	0.95	0.12		360	0.44	0.57	0.05

Also shown is the average number of individuals in each validation population per location (*n*G × L) and per location-by-season (*n*G × LS) for each validation approach

*PA* predictive ability, *PACC* prediction accuracy, *se.PACC* standard error of prediction accuracy

Predictive ability and accuracy for the second examined scenario of predicting into an environment for which no phenotypic information is available (Table 5, across location) was generally lower than for the first scenario, except for prediction into the Forlí location. The lowest prediction accuracy was observed for prediction of genomic breeding value at Prosser.

### Predicted G × E effects

**Fig. 7:**
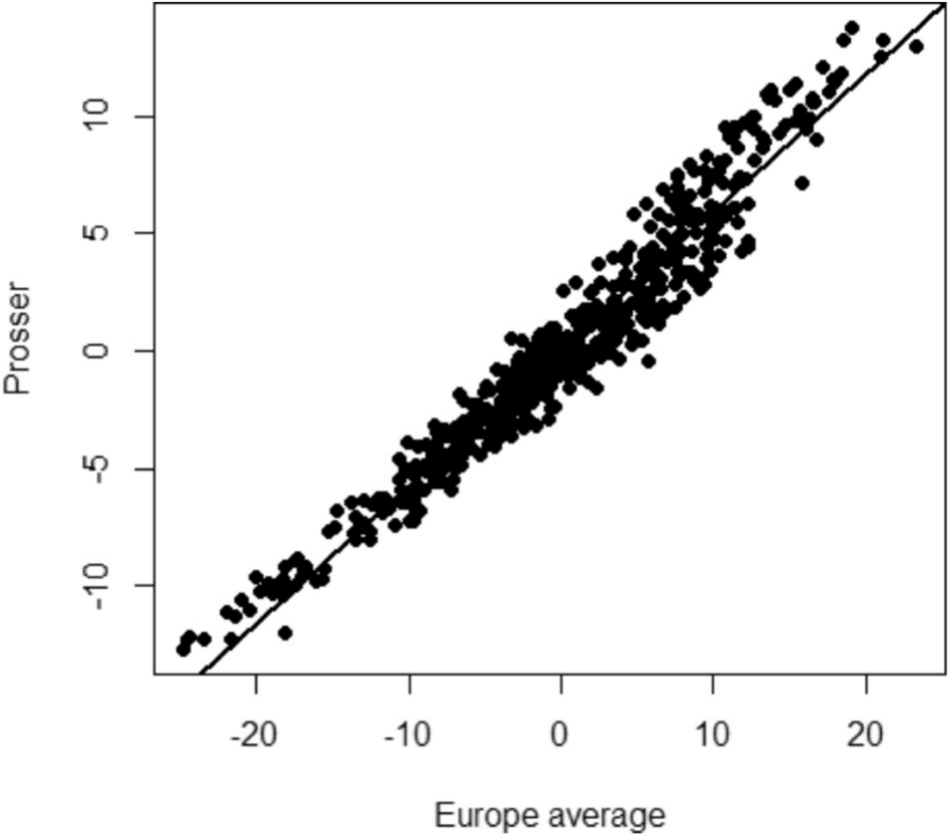
Scatter plot of genomic predicted breeding value of 550 individuals for fruit maturity timing (Julian days) averaged across three locations in Europe compared to genomic predicted breeding value in Prosser, USA

The high estimates of additive genomic correlation among environments supported accurate prediction of genomic breeding values for fruit maturity timing into the various environments for all individuals, even if they had not been phenotypically assessed in those environments (Fig. 7). As suggested by the high estimated additive genomic correlations between the European environments and Prosser, the relative ranking of predictions for individuals of average fruit maturity timing across the three European locations was largely consistent with the ranking at Prosser. The earliest predicted average fruit maturity timings across the three European locations were for ‘Precoce d’Isigny’, ‘Belle de St Denis’, ‘Abouriou’, ‘Guigne Précoce du Marché’ and ‘Scwecja’, which were 22–25 days earlier than the mean (30 May). Individuals predicted to the latest (19–23 days later than the mean) across the European locations were ‘Ambrunés’ and four selections from the Pacific Northwest Sweet Cherry Breeding Program. At Prosser, the five predicted earliest individuals were four of the five earliest across the European locations (all except ‘Scwecja’) and one selection from the US breeding program, which compared to the mean fruit maturity date for Prosser (2 July) were 12–13 days earlier. The five individuals predicted to be the latest in the European locations were also those predicted to be the latest at Prosser (13–14 days later than the mean date).

## Discussion

### Genetic structure of germplasm

The degree of decay in LD among loci with increasing physical distance across the sweet cherry germplasm in this study is comparable, but slightly weaker, than that reported for only the Bourran germplasm^40^. In this current study, the threshold for which LD was <0.2 occurred between 140 and 240 kb, suggesting slightly stronger LD than observed in Campoy et al.^40^ (0.2 threshold around 100 kb). The stronger LD in our study might be a consequence of the inclusion of full-sib families from the Prosser location. The degree of decay in LD with increasing distance observed in this study and that of Campoy et al.^40^ is similar to that recently reported in a Belgian apple collection^59^, but higher than that reported for peach (<0.2 between 800 and 1800 kb^60)^ and an earlier study in apple 1000 kb^61^. Campoy et al.^40^ suggested that a lower LD decay reported for peach is a consequence of the presence of self-compatibility in that crop leading to longer regions of homozygosity from inbreeding through selfing. Higher LD in the apple study^61^ was likely a consequence of small effective population size in the seven pedigree-connected full-sib families used.

The lack of obvious genetic structure among germplasm displayed as the first two eigenvalue loadings for the SNP-derived relationship matrix suggests that there is considerable identity of chromosome segments among germplasm evaluated in the four locations, with individuals being as related across locations as within the same location. These results are consistent with the hypothesis of a bottleneck during domestication of this crop^62^. Using the same genetic data for the Bourran population as used here, Campoy et al.^40^ identified two main groups of individuals representing landraces and bred cultivars. Further analyses decomposed the groups into nine clusters, with North American-derived cultivars in the Bourran collection allocated to only two of these clusters^40^. The observed wider diversity of the RosBREED germplasm is not surprising as individuals were deliberately chosen to maximise diversity by including germplasm that had not been commonly used previously in North American breeding programs along with standard North American cultivars and their available ancestors^42^.

The contrast in distribution of diagonals and off-diagonals between the SNP and pedigree-derived relationship matrices observed here has been discussed by others^63^. The SNP-derived relationship matrix (GRM) is able to capture unknown pedigree relationships, and heterogeneity in relationships due to Mendelian sampling, that is not possible using only pedigree information to estimate relationship coefficients ^36^. Differences between the pedigree-derived and SNP-derived relationship matrices might also be a consequence of incorrect or incomplete pedigree information or improper scaling. The high frequency of pedigree-derived pairwise relationship coefficients (off-diagonals) with a value of 0, compared to the more balanced distribution of realised relationships estimated from genotypic data, suggests that pedigree records are incomplete. Germplasm individuals are generally assumed to be unrelated in the base generation of the pedigree-derived relationship matrix, but they might instead be related and those relationships can be captured in the GRM. Estimation of the GRM is also dependent on the estimated allele frequency for each SNP^35^. Ideally, allele frequencies for the base population are needed, but they are difficult to obtain. Allele frequencies in our study were computed from available genotyped accessions and not from a base population, which could have resulted in improper scaling. Nonetheless, Wang et al.^64^ suggests with strong quality control (e.g., call rate ≥0.9, addressing parent-progeny conflicts, minor allele frequency >0.05, good pedigree depth), differences between the elements of matrices can be reduced.

The higher variance of the GRM diagonal and off-diagonal elements, compared to those for *A*, suggests predictions using a realised relationship matrix will be more accurate^36^. In this study in particular, the GRM might also more accurately estimate additive effects compared to a relationship matrix based on expected relationship coefficients derived from pedigree information, as there were only a small number of offspring per family and so there might have been insufficient replication of Mendelian sampling events within each family to accurately obtain a family average, which is an important source of information for prediction of genetic effects^65^.

Estimates >1 for the expected relationship coefficients of an individual with itself dervied  from pedigree information, and large realised relationship coefficients estimated from genotypic data, suggest that some individuals included in this study are inbred, as was also observed in western larch by, e.g., Klápste et al.^66^ This conclusion agrees with general knowledge of sweet cherry cultivar pedigrees, which involved a strong bottleneck, repeated use of a small number of parents, and crossing of individuals that shared recent ancestors^40,62^. For example, breeding programs have continuously used the cultivar ‘Stella’ and its descendants in breeding for self-compatibility, presumably dramatically reducing the genetic diversity of released cultivars^14^.

### Model fitting

This study employed a FA parameterisation to estimate parameters of the genetic-by-environment covariance matrix. While FA models have similarities with additive main effects multiplicative interactions (AMMI)^30,67^ approaches, AMMI is a fixed effects approach, whereas FA mixed models offer more flexibility such as fitting genotypes and genotype-by-environment interaction as random effects^30^. A mixed model approach more easily deals with unbalanced datasets and supports incorporation of heterogeneous variances and covariances among environments—all of which are essential features of our dataset. Superiority of FA models over AMMI models have been demonstrated using empirical MET eucalypt and teak datasets^68^.

Despite the high average additive genomic correlation among environments ($\bar r_{{\mathrm{A}} \times \mathrm{E}}$) estimated from a simple compound symmetry model, the FA G × E model was a significantly better fit to the data as it allowed heterogeneous variances among locations and heterogeneous pairwise covariances (and hence correlations) among locations. The compound symmetry structure constrains all variances and all covariances to be the same, which is an unrealistic assumption particularly when the environmental sampling is complex as demonstrated here. In contrast, the FA model captured the greater stability of genetic effects among and within the European locations compared to genetic effects at the Prosser location.

This study only applied an additive genetic effects model. Theoretically, inclusion of significant non-additive effects, if present, is expected to increase PACC and support selection for clonal value (i.e., total genetic effects = additive + non-additive genetic effects) where individuals can be vegetatively propagated^69–71^ as sweet cherry can be and is commercially. However, separating additive and non-additive genetic effects requires powerful designs or clonal replication of individuals. Moreover, fitting these effects separately in genomic prediction models have not shown meaningful improvement in accuracy, e.g. ref. ^72^, unless dominance was the predominant genetic effect and broad-sense heritability was >0.6^71^. Dominance effects for days to maturity have been estimated to compose a minor component of genetic variance in sweet cherry^11^.

The FA G × E model supported the combination of adjustment of phenotypes for fixed effects and estimation of genetic parameters into a multivariate single-step analysis. The realised relationship matrix was used to describe correlated genetic effects across environments so that data from various sources could be combined. Prediction accuracy from multivariate approaches is generally higher than that from univariate approaches and supports the prediction of genetic effects for individuals in environments in which they have not been tested by leveraging of correlated information^29,73,74^. In contrast to the single-step approach taken here, other studies have applied a two-stage analysis which  adjusts phenotypes for fixed effects prior to fitting of the genetic model, e.g., refs. ^36,72^. A single-step analysis as reported here was possible due to the small size of the population. Single-step analyses allow incorporation of complex models in the analysis^75^. While Welham et al.^76^ suggest that PACC from a single-step analysis is similar to that from a two-stage analysis, de los Campos et al.^77^ have reported that two-stage approaches for genomic prediction might produce biased genetic marker effects and correlation among residuals.

The estimates of PACC in all environments reported here are considerably higher than those reported previously for other traits in other horticultural tree crops^72,78,79^. The within-location validation strategy adopted here is similar to the second validation strategy adopted by Kumar et al.^72^ Here, training populations were constructed by excluding a subset of individuals within a location to simulate the strategy of predicting the performance of an individual within a target environment for which phenotypic data was available from other genotyped individuals. The high accuracy of this strategy (>0.8) might be explained in part by the high apparent heritability of fruit maturity timing (>0.60). The high prediction accuracies of the model for individuals at the Balandran and Bourran locations (>0.85) are also likely to be a consequence of the high genomic correlation (0.98), and large number of individuals replicated (68), between these locations. The high PACC for individuals at the Prosser location (>0.95) is likely to be a consequence of the random sampling strategy used to form the validation population as we did not account for the known complex family (and ancestral) structure. It is well known that close relationships between the training and validation populations increases PACC^80^. The lower accuracy of the across-location validation strategy of predicting across environments (0.50–0.95) is probably a consequence of the confounding of G × E with within-environment PACC.

### Implications of results for germplasm management

This study appears to be the first to quantify differences among locations in timing of fruit maturity in sweet cherry, particularly on a global scale. The advantage of undertaking a combined multivariate analysis like presented here is that location means are unbiased because they are adjusted for differences in germplasm composition among environments. In this way, comparisons are not confounded by the use of different germplasm in each environment. Differences among seasons within a location were not as great as differences between the European locations and Prosser, suggesting that fruit maturity across the European locations is generally earlier than at the Prosser location, at least for the seasons sampled here. More locations and seasons evaluated could be used to develop a more general predictive equation. A joint analysis of flowering timing and fruit maturity timing could help determine the extent of this phenological phenomenon.

High heritability estimates for timing of fruit maturity in sweet cherry have been reported previously^10^, suggesting that an individual’s breeding value for timing of fruit maturity is reliably predicted from the phenotype of that individual. However, our model only included additive genetic effects, and others^72,81^ have demonstrated that heritability estimates can be inflated if large non-additive effects are present but omitted from the training model. Deflated estimates of additive genetic variances in complex models might also be a consequence of correlation among additive and non-additive genetic effects^82^.

We are not aware of any previously published estimates of G × E for any traits, including fruit maturity timing, in cherry. Despite the detection of significant G × E in this study, G × E is somewhat low compared to the degree reported for most other traits in horticultural tree crops, e.g., ref. ^83^. Differences in ranking between seasons at Prosser was the major source of G × E detected in this study. The limited number of locations in this study prohibits the development and testing of a hypothesis to explain this pattern. The generally high correlation among all locations suggests that the timing of fruit maturity of an individual relative to other individuals is expected to be stable across production environments similar to these test locations. This conclusion is supported by the high correlation of predicted genetic values at Prosser with the average of predicted values across the three European locations (Fig. 7). Results also fit with the general observation that traits with high heritability exhibit low levels of G × E, which might be due in part to numerical constraints^22^.

The high narrow-sense heritability for fruit maturity timing reported here provides clear evidence of the large genetic gain that can be achieved by selecting for particular fruit maturity timing. The high genetic correlation among environments for this trait (>0.82) suggests that individuals can be selected for elite average performance across environments similar to those examined here, and elite germplasm in one environment for fruit maturity is expected to be elite in other environments. However, it is difficult to generalise this recommendation to new environments. In addition, other traits might exhibit different G × E patterns. However, the genetic potential of genotyped but untested individuals can be predicted using the methods outlined here if genomic and phenotypic data are available for other individuals and are used to estimate a prediction model.

### Extension of method

This study is based on a model of individual SNP loci in linkage disequilibrium with chromosome segments containing numerous small-effect QTLs such that replication of SNP alleles across environments models the replication of causal alleles at the small-effect QTLs. However, as previous studies^10,12^ have suggested the presence of large-effect QTLs contributing to genetic variation of fruit maturity timing, the model of genetic architecture used in this study might not be accurate. Incorporation of known QTL effects in the linear model should improve accuracy, as it is expected that such a model would more closely approximate the true distribution of QTL effects^77^. One of the challenges of building these models is that characterisation of the variation around QTL regions as individual biallelic SNPs might not capture the complexity of variation in the region. This variation might be better modelled using the same genotypic data by describing SNP haplotypes that contain QTL regions. The level of LD in cherry is sufficiently high that this information could be used to phase the SNP genotypes (for example, with Beagle^51^). Otherwise, the model could use separately obtained genotypic data that describes the allelic composition of each individual at large-effect QTLs with multiple effective alleles.

Incorporation of data from additional environments would be useful to develop a more general description of G × E patterns for the trait, enable development and testing of hypotheses to explain the G × E patterns, and increase PACC. Only a minor degree of G × E was detected in this study and thus there is insufficient variation to develop and test hypotheses of environmental drivers of G × E for fruit maturity timing. Increasing the environmental range might induce greater G × E. In this case, it might be that repeatable prediction of G × E could be achieved through incorporation of environmental variables or through use of crop growth models that associate variability in underlying physiological process with variation in environmental drivers^84,85^.

This study has demonstrated that genome-wide SNP-derived relationship matrices can be used to quantitatively describe cryptic genetic relationships. In addition, this study has demonstrated that such realised relationships can be used to combine phenotypic data collected on individuals evaluated across disparate locations into a single analysis without the need for clonal replication or knowledge of pedigree relationships. These approaches now allow the latent value of existing, otherwise unconnected, historical phenotypic datasets collected by numerous local breeding and cultivar testing programs to be exploited. Such analyses can increase knowledge of the genetic architecture of important traits to support ongoing genetic improvement, as well as having the immediate practical value of predicting genetic effects of individuals (i.e., breeding or clonal value) in untested environments. This type of analysis would be informative for other important horticultural traits of sweet cherry, such as bloom timing, productivity, fruit size, fruit firmness, fruit sweetness and fruit acidity, for which phenotypic data are largely available among breeders and cultivar testers.
